# Experimental Investigation of Solar-Driven Hollow Fiber Membrane Liquid Dehumidification System

**DOI:** 10.3390/membranes13040383

**Published:** 2023-03-27

**Authors:** Cai-Hang Liang, Jia-Li Hu, Nan-Feng Li, Zhi-Peng He, Chou Mo, Si Zeng

**Affiliations:** 1 Key Laboratory of Microelectronic Packaging and Assembly Technology of Guangxi Department of Education, School of Mechanical and Electrical Engineering, Guilin University of Electronic Technology, Guilin 541004, China; 2 Guangxi Beitou Environmental Protection & Water Group Co., Ltd., Nanning 530025, China

**Keywords:** hollow fiber membrane, liquid dehumidification, solar energy regeneration, experimental research

## Abstract

The hollow fiber membrane modules act as dehumidifiers and regenerators to avoid gas–liquid entrainment problems in direct-contact dehumidification systems. A solar-driven hollow fiber membrane dehumidification experimental rig was designed to investigate its performance from July to September in Guilin, China. The dehumidification, regeneration, and cooling performance of the system between 8:30 and 17:30 are analyzed. The energy utilization of the solar collector and system is investigated. The results show that solar radiation has a significant influence on the system. The hourly regeneration of the system has the same trend as the temperature of solar hot water, which ranges from 0.13 g/s to 0.36 g/s. The regeneration capacity of the dehumidification system is always larger than the dehumidification capacity after 10:30, which increases the solution concentration and the dehumidification performance. Further, it ensures stable system operation when the solar radiation is lower (15:30–17:50). In addition, the hourly dehumidification capacity and efficiency of the system ranges from 0.15 g/s to 0.23 g/s and 52.4 to 71.3%, respectively, with good dehumidification performance. The COP of the system and solar collector have the same trend, in which their maximum values are 0.874 and 0.634, respectively, with high energy utilization efficiency. The solar-driven hollow fiber membrane liquid dehumidification system performs better in regions with larger solar radiation.

## 1. Introduction

Carbon dioxide gas emissions are the main cause of global climate change, with serious impacts on the environment and human health. To address global climate change, countries around the world have adopted strict policies and measures to reduce carbon dioxide gas emissions. China has set a target to peak carbon emissions by 2030 and become carbon neutral by 2060 [1]. In 2020, the energy consumption of the global building and construction sector accounted for 36% of global final energy consumption and 37% energy-related carbon dioxide emissions, and air conditioning accounted for about 30% of energy consumption in the building and construction sector [2]. In China, air conditioning energy consumption accounts for about 50% in building energy consumption and 15% of the total energy consumption in 2020 [3]. In hot and humid climates, such as the coastal areas of southeastern China, the relative air relative is very high, sometimes up to 90% in summer. As a result, the air dehumidification load accounts for 20% to 40% of the total air conditioning load, and it can be even higher when 100% fresh air ventilation is required for indoor environmental control [4]. Therefore, an efficient and low carbon dehumidification technology is urgently needed for saving energy and reducing emissions. 

**Figure 1:**
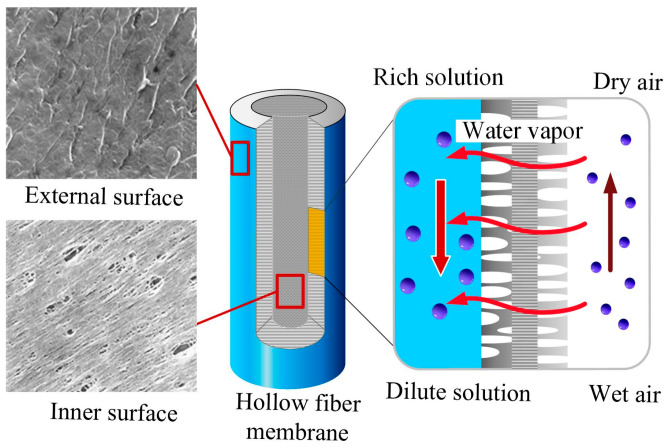
A schematic diagram of water separation.

With the development of membrane separation technology, membranes are widely used in water vapor separation. Water vapor can pass through the membrane, and the direction and transfer rate of water vapor are determined by the differential pressure of water vapor between the two sides of the membrane. A schematic diagram of water separation is shown in Figure 1. Organic polymer materials are the most widely used materials in water vapor separation due to their low price, easy reproducibility, and robustness [5]. The hydrophobic porous membranes, such as polypropylene (PP), polytetrafluoroethylene (PTFE), polyvinylidene fluoride (PVDF) and polyethylene (PE) for water vapor separation are widely used due to their lower moisture resistance [6,7,8,9,10].

In recent years, membrane liquid dehumidification as a water separation technique has emerged as an attractive alternative to conventional direct contact liquid dehumidification technology, due to its high efficiency, environmental friendliness, and so on. In 2014, a US Department of Energy technical report stated that membrane liquid dehumidification technology has the greatest potential to become the next generation of HVAC [11]. It has attracted increasing attention from academia and industry [12,13,14,15,16,17,18]. Compared with the conventional liquid dehumidification technology, the membrane liquid dehumidification adopts the membrane contactor to replace the packed-type dehumidifier. There are two main types of membrane dehumidifiers: flat membrane and hollow fiber membrane. The flat membrane dehumidifier has the advantages of simple structure and convenient assembly, but its filling density is low. The hollow fiber membrane has a highly specific surface area and better heat and mass transfer performance than the former. Because of its small size, it can be used in different modules. The system adopts a hollow fiber membrane module as a dehumidifier. In the system, liquid desiccant flows in the tube side, and air flows in the shell side. The system uses a semi-permeable membrane to separate the process air in the feed side from the liquid desiccant in the permeate side. The membranes used for the membrane liquid dehumidification can only permit moisture permeation, but they prevent liquid leakage [19,20]. The problem of liquid carryover of salt solution, which endangers human health and pollutes the indoor environment, is avoided [21,22]. The common dehumidification membranes are the microporous membrane, homogeneous membrane and composite membrane. The microporous membrane has a loose structure, with a pore size of 3100 nm, which the water molecule can easily pass through. The pore size of homogeneous membrane is generally below 1.5 nm, also called the compact membrane. With the thin hydrophobic membrane films as the support layer and the porous hydrophilic films as the top layer, the composite membrane prevents the wetting of the membrane and reduces overall transport resistance. Zhang developed a highly efficient semi-permeable membrane with hydrophilic/hydrophobic composite membrane for air dehumidification application [23,24,25]. The hydrophobic porous membranes are made of PVDF with low moisture resistance, high chemical stability and good mechanical strength. The hydrophilic membranes are a kind of ultra-thin dense membrane with high moisture absorption capacity. In addition, hydrophilic membranes have a high selectivity, allowing only water vapor to pass through and no other unwanted gases. The hydrophilic membranes are made of polyvinyl alcohol (PVA). The PVA/PVDF composite membrane used in this study was obtained from the group of Professor Lizhi Zhang at the South China University of Technology, China.

Solar energy is an essential source of renewable energy. Huang and Zhang [26] proposed a solar-powered hollow fiber membrane liquid dehumidification system. This system combines a solar hot water system with a hollow fiber membrane liquid dehumidification system. The system uses solar energy to generate hot water, to regenerate the salt solution. The system uses a hollow fiber membrane to replace the conventional absorber and regenerator to solve the liquid carryover problem for air liquid dehumidification as well. Therefore, it is a high-energy-efficiency and low-carbon dehumidification system. Khoudo et al. [27] studied the feasibility performance of using a solar-powered membrane liquid dehumidification system to remove humidity from an office space. The results showed that the system reduced 10% of indoor relative humidity, and achieved better indoor thermal comfort and better cost-effectiveness. Ahmed et al. [28] conducted the thermo-economic performance of a solar-powered hollow fiber membrane liquid dehumidification system. Compared with the conventional heat pump heat system and natural gas boiler heat system, solar thermal system has a higher initial investment cost, but the lowest operating and life-cycle costs. Bahaidaraha et al. [29] numerically conducted a solar-powered liquid dehumidification system using hollow fiber membrane and flat membrane dehumidifiers, respectively. The comparison results show that the hollow fiber dehumidifier has higher dehumidification efficiency than the flat membrane dehumidifier. Yin et al. [30] numerically investigated the energy-matching characteristics of solar-energy-driven membrane liquid desiccant system. They defined the energy-matching coefficient of the cooling and heating process of the solution, and analyzed the typical hot and humid climate conditions in southern China to obtain the parameters when the energy of the system reached its matching. Men et al. [31] developed an exergy analysis model of a solar-powered hollow fiber membrane liquid dehumidification system and analyze the exergy of the components. The effect of air and chilled water inlet parameters and solution flow rate on system performance was investigated. Gurubalan et al. [32] analyzed the energy-saving potential of solar-assisted membrane dehumidification systems in hot and humid regions. Compared to conventional air conditioning systems, the system saved approximately 62% of energy per year.

Many scholars have done some thermodynamic studies on membrane modules and liquid dehumidification systems, containing performance studies and optimization of dehumidification modules, and heat and mass transfer performance of liquid dehumidification systems with different membrane modules as dehumidifiers. Since the solar-driven hollow fiber membrane liquid dehumidification system was proposed, some scholars have carried out theoretical research on its thermodynamic performance. However, there are few experimental studies that have been conducted for this research system. The group designed and built a solar-driven hollow fiber membrane liquid dehumidification system experimental bench. The current work aims to experimentally study: (1) the system operation situation under actual climatological conditions; (2) dehumidification performance and regeneration performance of the system to reveal the thermodynamic properties of the system more deeply; and (3) energy utilization rate of the system and evaluating the feasibility of the system in hot and humid regions.

## 2. Experimental Set-Up

The system mainly consists of a hollow fiber membrane liquid dehumidification system, a solar water heating system, and a cooling water system. The thermal driving force of the dehumidification system comes from the solar collector, which provides regenerative heat to regenerate the dilute salt solution using solar energy. The air-cooled chiller provides cooling water to decrease the temperature of the rich solution after regeneration.

### 2.1. Hollow Fiber Membrane Liquid Dehumidification System

**Figure 2:**
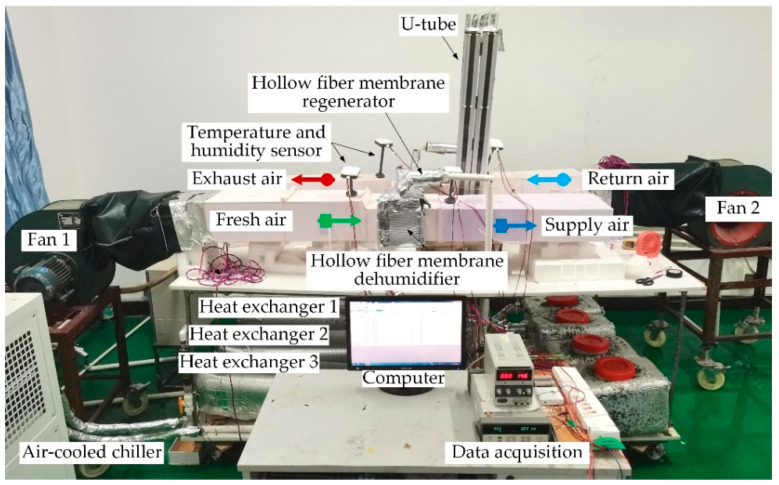
The hollow fiber membrane dehumidification system experimental rig.

An experimental rig has been designed to investigate the performance of the hollow fiber membrane dehumidification system, shown in Figure 2. The dehumidification system consists of two hollow fiber modules, three heat exchangers, a solution tank, three pumps, and two fans. The pumps and fans supply circulation power for fluids in the dehumidification system, while the heat exchanger regulates the solution temperature at the inlet and outlet of the membrane module. The humidification and regeneration modules have the same structure, which is the core structure of the whole dehumidification system. The dehumidification and regeneration modules perform air dehumidification and solution regeneration functions, respectively.

**Figure 3:**
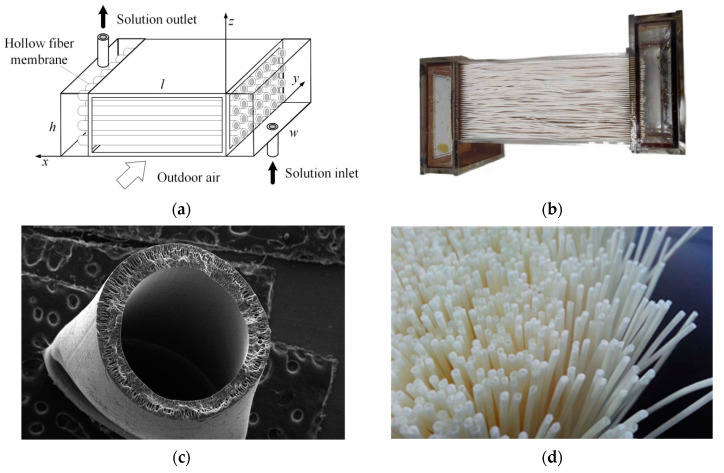
Hollow fiber membrane module: (**a**) schematic diagram; (**b**) structure; (**c**) microstructures; (**d**) physical objects.

The schematic diagram and structure of the membrane module are shown in Figure 3a,b. The geometry structures and membrane properties of the hollow membrane module are listed in Table 1. The structure of the hollow fiber membrane module is similar to the shell-and-tube heat exchanger, but the metal tubes are replaced with hollow fiber membranes that allow simultaneous heat and moisture transfer between the air and salt solution. The membrane module consists of a series of porous moisture semi-permeable membranes with high selectivity and permeability, of which the microstructures and physical objects are shown in Figure 3c,d. The solution flows in the hollow fiber membrane tube, and the air flows across the hollow fiber membrane tubes. The direction of the air stream is perpendicular to the direction of the solution stream, and the tube bundle is arranged in an equilateral triangular form. This study used a 35% lithium chloride (LiCl) solution concentration to reduce the crystallization risk and obtain better dehumidification efficiency.

**Figure 4:**
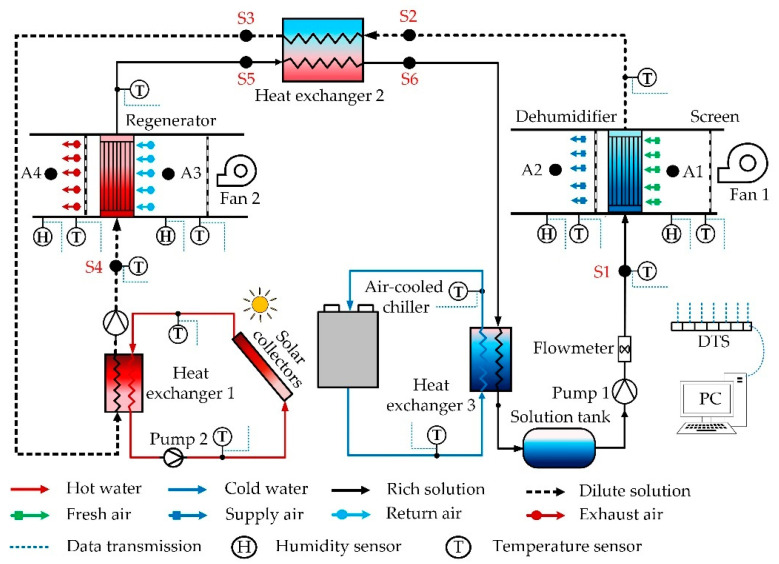
Schematic diagram of the hollow membrane liquid dehumidifier system.

**Figure 5:**
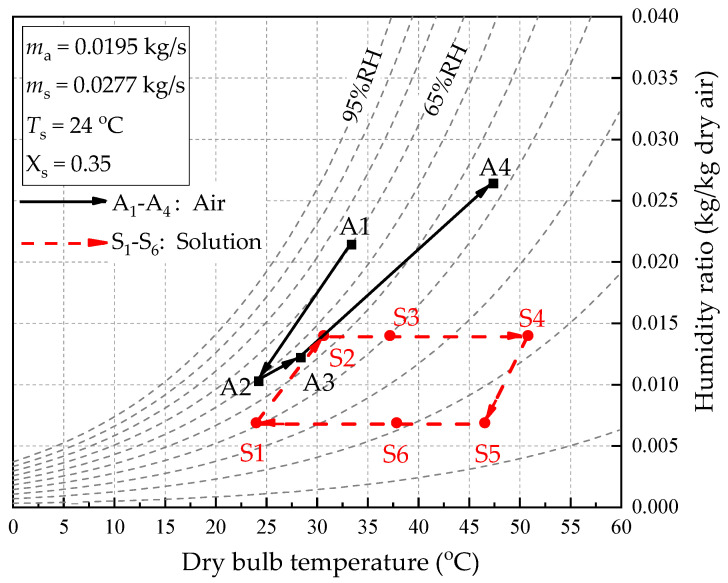
The psychrometric diagram of the air and solution streams in the dehumidifier system.

The schematic diagram of the hollow fiber membrane dehumidifier system is shown in Figure 4. In the system, the fresh air (A1) is blown into the dehumidifier by a fan and contacts the desiccant liquid in the tube indirectly. Because the water vapor partial pressure of the air outside the membrane is greater than that of the solution in the membrane, the moisture in the air permeates through the hollow fiber membrane into the desiccant liquid. Hence, the fresh air is dehumidified (A2) and supplied into the room. The exhaust air (A3) flows through the regenerator to absorb the moisture in the desiccant liquid, and then the air becomes hot, and humid air (A4) exits. In the LiCl solution circuit, the rich solution (S1) is taken from the solution tank and sent to the dehumidifier at low temperature. The rich solution flows through the inner fiber tubes in the dehumidifier and absorbs the moisture in the fresh air. The low-temperature dilute solution (S2) first flows the through solution–solution heat exchanger (HE2) and exchanges heat with the high-temperature rich solution (S5). In the heat exchanger, the dilute solution is heated (S3), and the rich solution is cooled (S6). The hot dilute solution (S3) flows through the hot water–solution heat exchanger (HE1) and exchanges heat with hot water heated by a solar collector. The solution temperature is further increased (S4) and sent to the hollow fiber membrane regenerator. In the regenerator, the water vapor partial pressure of the solution is greater than the exhaust air, so the moisture in the solution is absorbed by the exhaust air, and the high-temperature dilute solution (S4) is regenerated in the rich solution (S5). After cooling in the HE2, the rich solution (S6) flows into the solution–cold-water heat exchanger (HE3) for further cooling (S1) and storage in the solution tank. The tank acts as a cold storage device to store the low-temperature concentrated solution for the next cycle. The psychrometric diagram of the air and solution of the system is shown in Figure 5.

### 2.2. Solar Water Heating System

**Figure 6:**
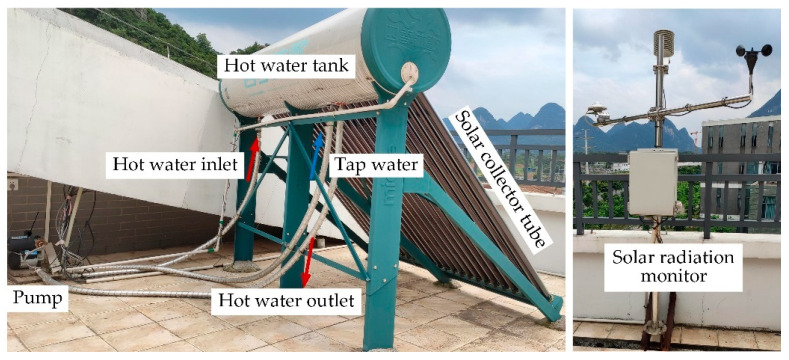
Solar water heating system.

The regeneration of the liquid desiccant is critical for maintaining the continuous operation of the system. The hot water used to regenerate the dilute solution is provided by the solar heating system. The main components of the solar water heating system are the solar collector, heat storage tank, water pump, hot-water–solution heat exchanger, and solar radiation monitoring instrument, as shown in Figure 6. The solar collector absorbs and collects solar radiation to transfer heat to the water in the tank. The tank supplies hot water to exchange heat with the desiccant solution in the heat exchanger. The liquid desiccant temperature is raised to achieve a better regeneration effect.

The solar collector is the core component of the solar heating unit, which consists of 24 vacuum tubes. The effective surface area of the solar collector tube is 3.0 m^2^. The hot water tank is the main heat storage part of the heating unit. The tank volume is 180 L, and the thickness of the insulation layer is 57 mm. The pump, with a head of 25 m, is used to return hot water to the storage tank. The solar radiation monitoring instrument is used to collect real-time solar radiation, ambient air temperature and humidity.

The temperature of each key position of the solar heating unit is measured by thermocouples, while the volumetric flow rate of water is measured by glass flow meters. In addition, the solar radiation amount is measured by a radiance meter with a sensitivity of 10.73 μV/(W·m^2^).

The system was operated from 8:30 to 17:30 from July to September 2020 in Guilin, China, a representative region of a hot and humid climate. The data are read and recorded every 10 min after the system runs stably. Steady-state conditions are reached in approximately 30 min.

### 2.3. Cooling Water System

**Figure 7:**
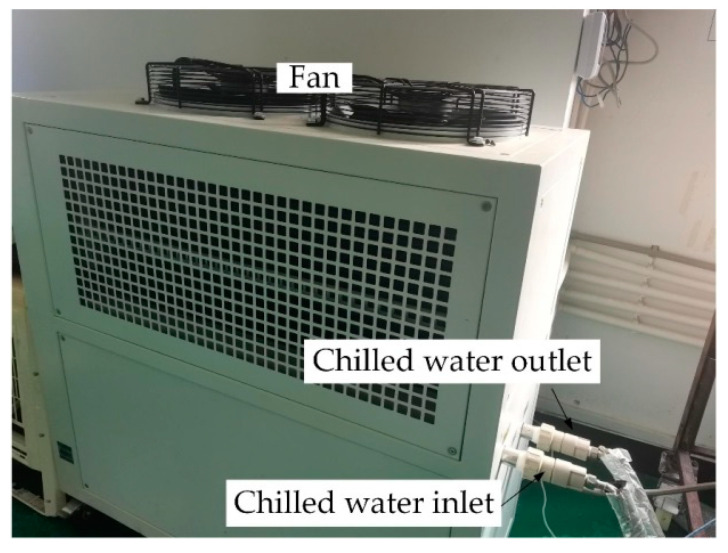
Cooling water system.

In this study, a FUKEL FIC-3A air-cooled chiller is employed to provide the cooling capacity for the solar-driven hollow fiber membrane liquid dehumidification system, as shown in Figure 7, which has a total power of 2.8 kW. The chiller has an accuracy of ±1 °C and can provide cooling water from 4–45 °C, which satisfies the cooling water temperature demanded by the dehumidification system (18–22 °C). For regions with an annual average temperature below 15 °C, natural cold sources such as deep groundwater and soil, can provide sufficient cooling capacity. However, mechanical cooling is necessary to maintain the stable operation of dehumidification systems in regions with high average annual temperatures, such as Guilin, China. The regenerated rich solution has a higher temperature and cannot participate directly in the system circulation. Based on this fact, the solution needs to be fed into the heat exchanger 2 for pre-cooling and then into heat exchanger 3 for heat exchange with the cooling water. The solution temperature decreases significantly. The lower the temperature of the solution, the lower its water vapor partial pressure, which can enhance the dehumidification performance effectively. Meanwhile, the heat transfer performance is enhanced as the temperature decreases.

### 2.4. Experimental Procedure

The detailed operation steps of the experimental system are as follows:

(1) Prepare a 35% LiCl solution with LiCl powder and deionized water;
(2) Fill the solution tank with 35% LiCl solution to the operating level;
(3) Turn on the solar collector, adjust the hot water flow rate and run for a period of time, until the hot water temperature meets the regeneration temperature;
(4) Turn on the air-cooled chiller and set the flow rate and temperature, until the chilled water temperature reaches a predefined value;
(5) Turn on the dehumidification and regeneration fans and adjust the air flow rate;
(6) Turn on the solution pump and adjust the solution flow rate to send the desiccant liquid from the solution tank to the dehumidifier;
(7) The system starts to run, and the experiment can be performed after the system parameters meet the requirements and stability.

After the dehumidification system is stabilized, experimental work will be performed based on the nominal parameters listed in Table 2. Among them, the hot water temperature is determined by the solar radiation received by the solar collector, which is a variable value. In addition, the ambient temperature and humidity ratio trends are similar to that of solar radiation, which shows an increase followed by a decrease. The ambient temperature increases as the solar radiation increases. Meanwhile, the soil, plants and water surface constantly transport water vapor into the environment due to evaporation, which increases the environmental humidity ratio. However, the ambient temperature and humidity ratio have the opposite pattern of change when solar radiation decreases.

## 3. Data Processing

### 3.1. Data Recording

The temperature and relative humidity of the air stream is measured by the Siemens QFM 2160 thermo-hygrometer, while the PT-100 temperature sensor meters the solution temperature. The water flow rate is measured by the LZB-10 flow meter, while the air flow rate is obtained by Testo 425 anemometer measurements and channel cross-sectional area calculations. The pressure drop can be obtained by the U-tube pressure gauges. The Agilent 34972A data acquisition device collects and records all the data, which saves the data in a computer at a user-specified time interval (e.g., every 10 min). All instruments are calibrated. The parameters of the experimental instrument are shown in Table 3.

### 3.2. Performance Indices

The dehumidification capacity (Δ*m*_deh_) means the mass of water vapor removed from the air stream by the desiccant liquid in the dehumidifier, which can be expressed as

(1) $$\Delta m_{\;\mathrm{deh}}=m_{\;\mathrm{deh},a}\left(\omega_{\mathrm{deh},\mathrm{ai}}-\omega_{\mathrm{deh},\mathrm{ao}}\right)$$

where *m*_deh,a_ is the air mass flow rate in the dehumidifier, kg/s; *ω* is the humidity ratio of the air stream, g water vapor/kg dry air; subscripts “ai” and “ao” mean inlet and outlet of the air stream.

The regeneration capacity (Δ*m*_reg_) represents the mass of water vapor carried away from the desiccant liquid by the air stream in the regenerator, which can be written as

(2) $$\Delta m_{\;\mathrm{reg}}=m_{\;\mathrm{reg},a}\left(\omega_{\mathrm{reg},\mathrm{ai}}-\omega_{\mathrm{reg},\mathrm{ao}}\right)$$

where *m*_reg,a_ is the air mass flow rate in the regenerator, kg/s; subscripts “deh” and “reg” denote the dehumidifier and regenerator.

The dehumidification efficiency (*ε*_deh_) is defined as the ratio of the actual humidity difference to the maximum humidity difference, which can be calculated by

(3) $$\varepsilon_{\mathrm{deh}}=\frac{\omega_{\mathrm{deh},\mathrm{ai}}-\omega_{\mathrm{deh},\mathrm{ao}}}{\omega_{\mathrm{deh},\mathrm{ai}}-\omega_{\mathrm{deh},\mathrm{si}}}$$

The total system cooling capacity (*Q*_total_) is defined as the energy change of the air stream, which can be obtained by

(4) $$Q_{\mathrm{total}}=m_{\mathrm{deh},a}\left(h_{\mathrm{deh},\mathrm{ai}}-h_{\mathrm{deh},\mathrm{ao}}\right)$$

where *h* is the enthalpy of the air stream, kJ/kg.

The heat transfer capacity of water during regeneration is calculated by the following equation:

(5) $$Q_{\mathrm{solar}}=m_{h,w}c_{p,w}\left(T_{h,\mathrm{wo}}-T_{h,\mathrm{wi}}\right)$$

where *m*_h,w_ is the mass flow rate of the hot water, kg/s; *c*_p,w_ is the specific heat capacity of the water stream, kJ/(kg·K); *T*_h_ is the temperature of the hot water, K; subscripts “wi” and “wo” mean inlet and outlet of the hot water.

The system coefficient of performance (COP_system_) can be calculated by

(6) $${\mathrm{COP}}_{\mathrm{system}}=\frac{Q_{\mathrm{total}}}{Q_{\mathrm{solar}}+Q_{c}}$$

where *Q*_c_ is the power consumption of the chillers, kW.

The coefficient of performance of the solar collector (COP_solar_) can be written as

(7) $${\mathrm{COP}}_{\mathrm{solar}}{=\mathrm{COP}}_{\mathrm{system}}\times \eta_{\mathrm{solar},c}$$

where *η*_solar,c_ is the solar collector efficiency, which can be calculated by

(8) $$\eta_{\mathrm{solar},c}=\frac{Q_{\mathrm{solar}}}{I\cdot A_{c}}$$

where *I* is the solar irradiance, W/m^2^; *A*_c_ is total area of the solar collector, m^2^.

### 3.3. Uncertainty Analysis

All of the sensors used for parameter measurements are calibrated to reduce errors. The indirect measurement parameters, such as the dehumidification capacity, regeneration capacity, efficiency and COP, cannot be measured directly. Therefore, an error propagation computation is necessary. The uncertainty analysis of the experimental results can be calculated as

(9) $$Y=f\left(x_{1},x_{2},\ldots ,x_{n}\right)$$

(10) $$\delta Y=\sqrt{{\left(\frac{\partial Y}{\partial x_{1}}\delta x_{1}\right)}^{2}+{\left(\frac{\partial Y}{\partial x_{2}}\delta x_{2}\right)}^{2}+\ldots +{\left(\frac{\partial Y}{\partial x_{n}}\delta x_{n}\right)}^{2}}$$

where *x*_1_, *x*_2_, …, *x*_n_ are the parameters directly measured by the experimental instruments; *Y* is the function of the parameters; *δx*_1_, *δx*_2_, …, *δx*_n_ are the overall uncertainty of the parameters; *∂Y*/*x*_1_, *∂Y*/*x*_2_, …, *∂Y*/*x*_n_ are the relative errors of the function *f*; *δY* is the formula for error propagation; *δY*/*Y* is the overall uncertainty of the results. In this study, Δ*m*_deh_ uncertainty is 2.2%; Δ*m*_reg_ uncertainty is 5.3%; *Q*_total_ uncertainty is 4.6%; COP uncertainty is 4.1%.

## 4. Results and Discussion

### 4.1. Daily and Hourly Variations in Solar Radiation and Solar Collector Performance

**Figure 8:**
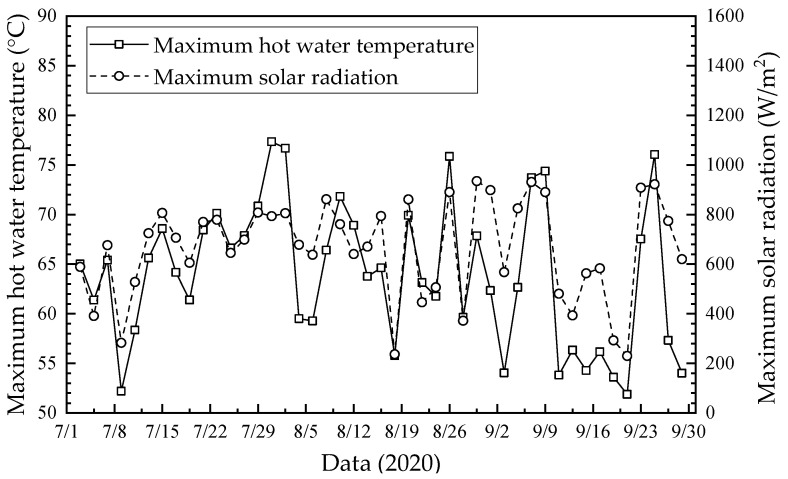
Daily maximum hot water temperature and solar radiation from July to September 2020.

The daily maximum hot water temperature and solar radiation from July to September 2020 are shown in Figure 8. As seen, the higher hot water temperatures usually correspond to larger solar radiation, while the lower hot water temperatures correspond to smaller solar radiation. The daily maximum solar radiation was in the range of 400 W/m^2^–1000 W/m^2^, except for some cloudy and rainy days, during which the daily maximum solar radiation was in the range of 100 W/m^2^–400 W/m^2^. In addition, the daily maximum hot water temperature was larger than 50 °C. 

**Figure 9:**
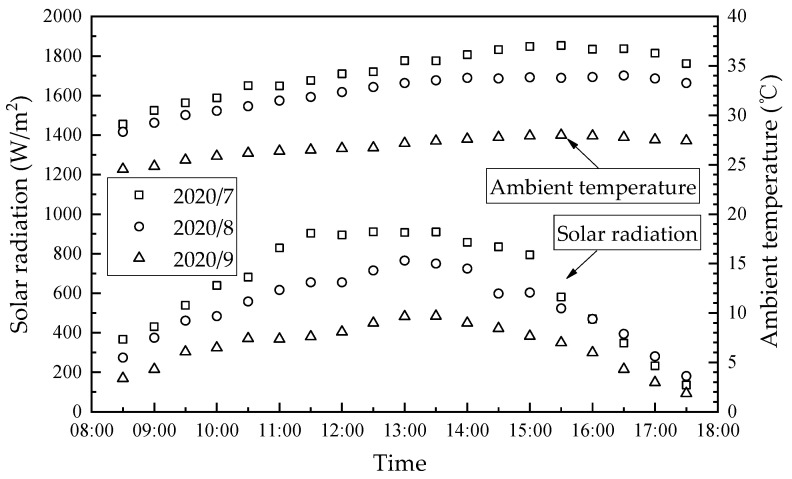
Hourly variation in average solar radiation and ambient temperature from July to September 2020 in Guilin, China.

The hourly variations of average solar radiation and ambient temperature from July to September 2020 in Guilin, China, are shown in Figure 9. The ambient air relative humidity and air velocity during the experiment were in the range of 50–80% and 1 m/s–4 m/s, respectively. As seen, the average solar radiation and ambient temperature were the highest in July and lowest in September. The solar radiation shows a trend of increase first and then decrease, which kept a high level at 12:30–13:30 and had a maximum value of 910 W/m^2^ at 11:30. Ambient temperature trends are more moderate than solar radiation, with the daily maximum temperature difference within 7.9 °C. The ambient temperature had a maximum value of 37.0 °C at 15:30, which had a lag time of 2–4 h compared to the maximum solar radiation.

**Figure 10:**
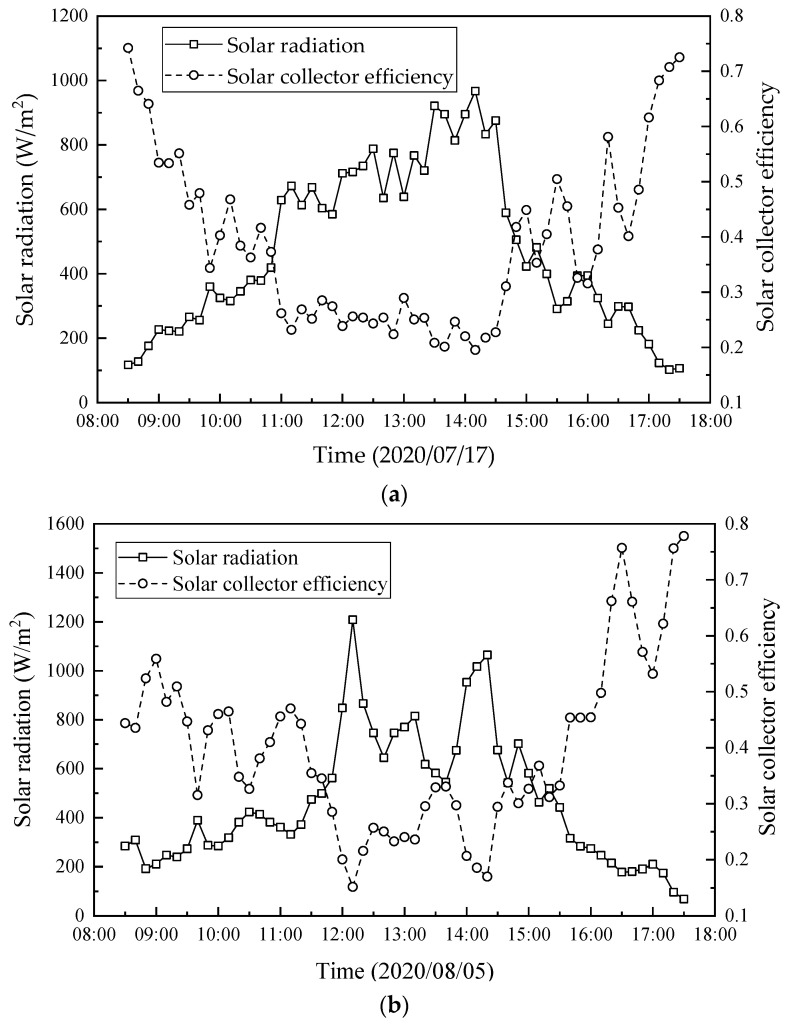
Hourly variation in solar radiation and solar collector efficiency on two days in July and August 2020: (**a**) 17 July 2020; (**b**) 5 August 2020.

The hourly variations of the solar radiation and solar collector efficiency on two days in July and August 2020 are shown in Figure 10. As seen, the solar collector efficiency presents a trend of decrease first and then increase, which is opposite to the solar radiation. It is because the heat absorbed by the water inside the solar collector was limited, while the unutilized heat was emitted to the ambient. Further, the heat absorbed by the solar collector increased with the increase in solar radiation, which was less than the increase in solar radiation. Therefore, according to Equation (8), the solar collector efficiency decreases with the increase in solar radiation.

### 4.2. Hourly Variation in Dehumidification Efficiency and Cooling Capacity

**Figure 11:**
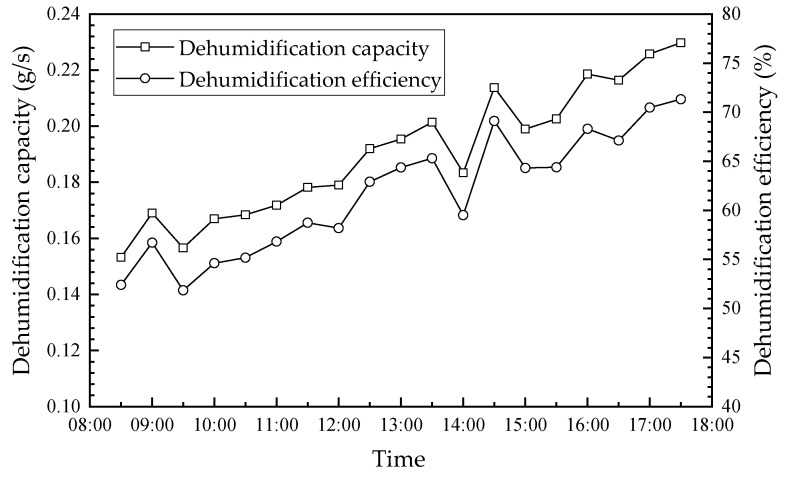
Hourly variation in the dehumidification capacity and efficiency.

**Figure 12:**
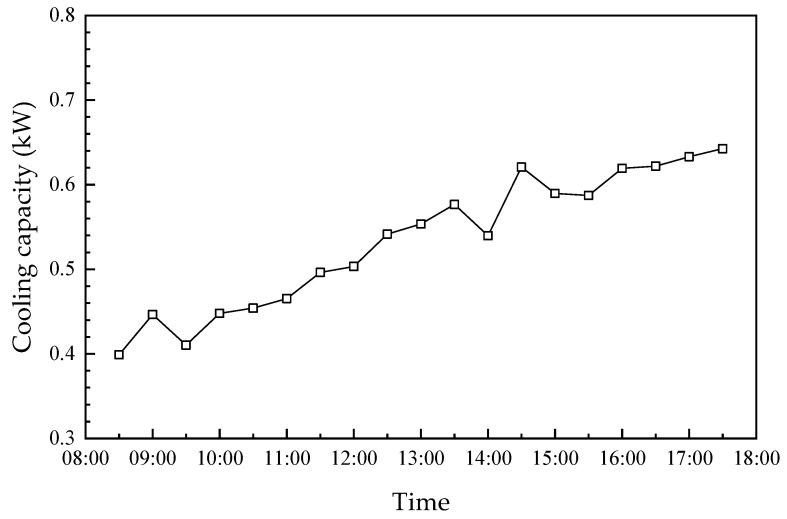
Hourly variation in the cooling capacity.

The fresh outdoor air was dehumidified and cooled before being sent into the room. The hourly variations in the dehumidification capacity, dehumidification efficiency and cooling capacity of the humidifier module are shown in Figure 11 and Figure 12. As seen, the dehumidification capacity, dehumidification efficiency and cooling capacity have similar trends. Further, their values increase from 0.15 g/s, 52.4%, and 0.4 kW to 0.23 g/s, 71.3% and 0.63 kW, from 8:30 to 17:30, respectively. Further, their values increased by approximately 53.3%, 18.9% and 57.5%, respectively. This is attributed to the following reasons: (1) the temperature/humidity difference is the driving force for heat/moisture transfer across the membrane, which increases with the increase in ambient temperature and humidity ratio. As mentioned in Figure 8, the ambient temperature increased first and then decreased during the day, which decreased slightly after 15:30. The dehumidification capacity, dehumidification efficiency, and cooling capacity increased accordingly. (2) The increase in hot water temperature increased the concentration of the solution after regeneration, which is beneficial to air dehumidification. The regeneration capacity was always larger than the dehumidification capacity between 12:00 and 16:30, which resulted in the solution always remaining at a higher concentration. Therefore, the dehumidification and cooling capacity of the system continuously increased. The hot water temperature inside the solar collector had a similar trend to the ambient temperature, which is described in detail in Section 4.3.

### 4.3. Hourly Variation in Hot Water Temperature and Regeneration Capacity

**Figure 13:**
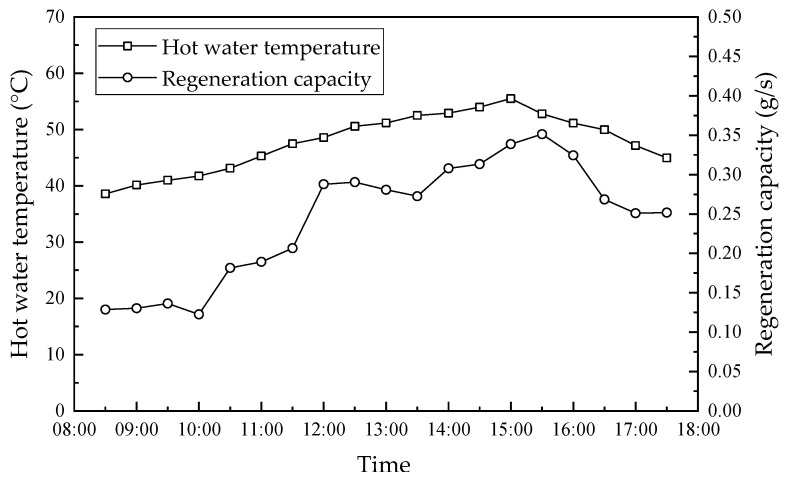
Hourly variation in hot water temperature and regeneration capacity.

The exhaust gas was used as the regeneration gas, which raised the solution concentration by removing the moisture from the dilute solution. Figure 13 shows the hourly variation in hot water temperature and regeneration capacity. As mentioned, the maximum solar radiation was approximately 910 W/m^2^ at 11:30, while the maximum hot water temperature was 55.5 °C at 15:00, which is because of the 1.5–3.5 h lag in the change of the hot water temperature within the solar collector. In addition, the hot water within the solar collector supplied the heat consumed for the dilute solution regeneration in the regenerator module. Therefore, the regeneration rate had a similar trend to the hot water temperature, which had a maximum value of 0.36 g/s at 15:30. The dilute solution temperature increased with an increase in the hot water temperature, which increased the vapor partial pressure of the dilute solution. Therefore, the vapor partial pressure and temperature difference between the two sides of the membrane increased, which resulted in more water vapor and heat transfer through the membrane to the air side. The regeneration efficiency increased consequently. The hot water temperature was higher than 48 °C after 12:00, while the regeneration capacity was in the range of 0.25 g/s–0.36 g/s, which was always larger than the maximum dehumidification capacity of 0.23 g/s. This means that the performance of the regenerator met the system cycle requirements. However, the regeneration capacity before 10:30 was less than the dehumidification capacity, which means that the dilute solution needed to be auxiliary-heated to maintain the steady operation of the system.

### 4.4. Hourly Variation in the COP of the Dehumidification System and Solar Collector

**Figure 14:**
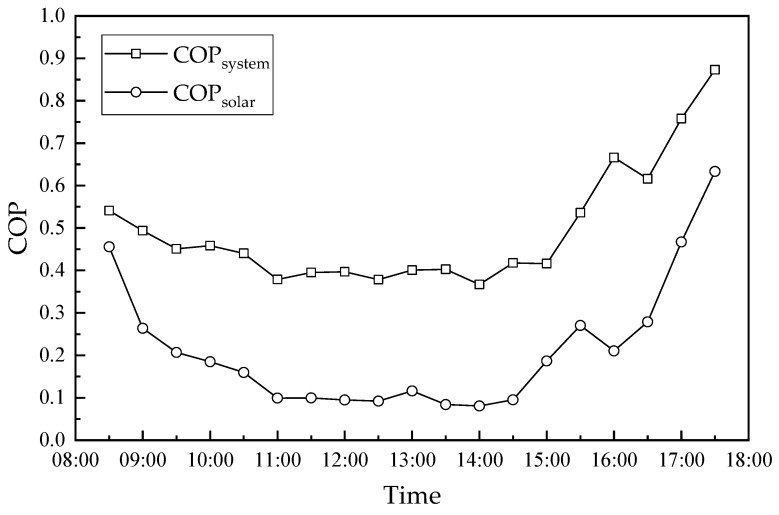
Hourly variation in the COP_system_ and COP_solar_.

The hourly variations in the COP of the dehumidification system and the solar collector are shown in Figure 14. As seen in Figure 8 and Figure 13, the COP of the dehumidification system and solar collector had the opposite trend to solar radiation. Further, they show a trend of decrease first, then stabilization for some time and then an increase, in which the COP of solar collectors changed more significantly. This is because the heat absorption performance of solar collectors was limited, while the heat not absorbed escapes into the ambient, which decreases the COP of solar collectors with the increase in solar radiation. In the first stage (8:30–11:00), the increased solar radiation resulted in more heat escaping into the ambient, so the COP of the dehumidification system and solar collector decreased from 0.54 and 0.46 to 0.38 and 0.099, respectively. In the second stage (11:00–14:30), solar radiation was maintained at a high level, while the COP of the dehumidification system and solar collector fluctuated in the range of 0.38–0.42 and 0.08–0.12, respectively. In the third stage (14:30–17:30), the performance coefficients of the dehumidification system and solar collector increased with the decrease in solar radiation, with their values increasing from 0.42 and 0.08 to 0.87 and 0.63, respectively. The COP of the dehumidification system and solar collector had the lowest values of 0.37 and 0.08 at 14:00, respectively. Meanwhile, their maximum values were 0.87 and 0.63 at 17:30, respectively.

## 5. Conclusions

In this paper, a solar-driven hollow fiber membrane dehumidifier experimental rig was designed to investigate its performance from July to September 2020 in Guilin, China. The solar radiation and hot water temperature in the solar collector during the experiment were recorded in real-time. The hourly variation in the solar collector, humidifier, regenerator and system performance was studied for two representative days between July and September 2020. The main conclusions drawn from this study are summarized as follows:

(1) The higher/lower hot water temperatures usually correspond to the greater/lower solar radiation. From July to September 2020 in Guilin, China, the maximum daily solar radiation was mainly in the range of 400 W/m^2^–1000 W/m^2^, while the maximum daily water temperature was between 50 °C and 80 °C. This means that the solar collectors provide sufficient energy to meet the regeneration demands of the dehumidification system and maintain the system operating continuously.
(2) The dehumidification capacity, dehumidification efficiency and cooling capacity had the maximal values of 0.23 g/s, 71.3%, and 0.63 kW at 17:30, respectively. The regeneration capacity between 12:00 and 16:00 was larger than the dehumidification capacity, while the desiccant solution concentration in the system kept increasing. Therefore, the dehumidification and cooling capacity increased accordingly. This provides for the performance of the dehumidification system when the solar radiation was lower (15:30–17:50).
(3) The hot water in the solar collector supplied energy for solution regeneration. The hot water temperature had a maximum value of 55.5 °C at 15:00, which lagged by about 1.5–3.5 h compared to the maximum solar radiation. The maximum regeneration capacity was 0.36 g/s, which was 0.13 g/s larger than the dehumidification capacity. The water temperature was above 48 °C after 12:00. The regeneration capacity was larger than the dehumidification capacity, which means that the regenerator performance met the system circulation demand. However, the regeneration capacity before 10:30 was less than the dehumidification capacity, which means that the dilute solution needed to be auxiliary-heated to maintain the steady operation of the system.
(4) Because the heat absorption performance of solar collectors was limited, the heat not absorbed dissipated into the ambient, which decreased the COP of solar collectors with the increase in solar radiation. Further, the COP of the dehumidification system and solar collector had the opposite trend to solar radiation. Further, they showed a trend of decrease first, then stabilization for some time and then increased, in which the COP of solar collectors changed more significantly.
(5) It is efficient and economical to supply regenerative energy to hollow fiber membrane liquid dehumidification systems by solar energy. The heat provided by the solar collectors can satisfy the system regeneration demands and ensure the stable operation of the system. However, the auxiliary heating of the regeneration solution is needed to provide regeneration heat when the solar radiation is lower, such as on cloudy and rainy days. In addition, the solar-driven hollow fiber membrane liquid dehumidification system performs better in regions with larger solar radiation.

## Figures and Tables

**Table 1:** Geometry structures and membrane properties of the hollow membrane module.

Geometry Structures	Values	Unit	Membrane Properties	Values	Unit
Module length	280	mm	Outer diameter	1.7	mm
Module width	150	mm	Inner diameter	1.5	mm
Module height	100	mm	Water contact angle	72 (outer)	°
				105 (inner)	
Tube pitch	3.5	mm	Pore size	0.10	μm
Packing Fraction	0.251	-	Diffusivity	9 × 10^−7^	m^2^/s
Number of total fibers	1660	-	Thermal conductivity	0.17	W/(m·K)

**Table 2:** Operating conditions of the hollow fiber membrane module.

Dehumidifier Module	Values	Unit	Regenerator Module	Values	Unit
Air flow rate	60	m^3^/h	Air flow rate	60	m^3^/h
Inlet air temperature	26.8–33.7	°C	Inlet air temperature	24.8–28.9	°C
Inlet air humidity ratio	19.2–21.5	g/kg	Inlet air humidity ratio	12.8–14.3	g/kg
Cooling water flow rate	120	L/h	Hot water flow rate	120	L/h
Cooling water temperature	22	°C	Solution flow rate	120	L/h

**Table 3:** Parameters of the experimental instrument.

Parameter	Model	Accuracy	Measurement Range
Air velocity	Testo 425	±0.03 m/s	0–20 m/s
Water flow rate	LZB-10	±4 L/h	16–160 L/h
Solution temperature	PT-100	±0.15 °C	−20–250 °C
Air temperature	Siemens QFM 2160	±3.00%	0–100%
Relative humidity	Siemens QFM 2160	±1 °C	−35–50 °C
Data acquisition	Agilent 34970A	±0.15%	-

## Data Availability

Not applicable.
